# Dietary intakes of fat and total mortality among Japanese populations with a low fat intake: the Japan Collaborative Cohort (JACC) Study

**DOI:** 10.1186/1743-7075-11-12

**Published:** 2014-03-06

**Authors:** Kenji Wakai, Mariko Naito, Chigusa Date, Hiroyasu Iso, Akiko Tamakoshi

**Affiliations:** 1 Department of Preventive Medicine, Nagoya University Graduate School of Medicine, 65 Tsurumai-cho, Showa-ku, Nagoya, Aichi 466-8550, Japan; 2 Department of Food Science and Nutrition, School of Human Science and Environment, University of Hyogo, 1-1-12 Shinzaike-Honcho, Himeji, Hyogo 670-0092, Japan; 3 Public Health, Department of Social and Environmental Medicine, Osaka University Graduate School of Medicine, 2-2 Yamadaoka, Suita, Osaka 565-0871, Japan; 4 Department of Public Health, Hokkaido University Graduate School of Medicine, N15 W7, Kita-ku, Sapporo, Hokkaido 060-0812, Japan

**Keywords:** Fat, Fatty acids, Saturated fatty acids, Monounsaturated fatty acids, Polyunsaturated fatty acids, Total mortality, Cohort studies, Japan

## Abstract

**Background:**

It may be useful to examine associations of fat intakes with total mortality as a basis for dietary recommendations. We aimed to elucidate associations between dietary fat and total mortality among Japanese populations with low fat intake.

**Methods:**

We conducted a prospective study consisting of 58,672 men and women aged 40 to 79 years. Fat intakes were estimated using a food frequency questionnaire. Multivariate-adjusted hazard ratios (HRs) for mortality by sex were computed according to quintiles of energy-adjusted fat intakes.

**Results:**

During the follow-up period (median duration, 19.3 years), 11,656 deaths were recorded. In men, we found no clear association between total fat and total mortality. HRs across quintiles of total fat intake were 1.00, 1.03 (95% confidence interval [CI], 0.95–1.12), 1.02 (0.94–1.10), 0.98 (0.90–1.07), and 1.07 (0.98–1.17). No significant association was detected in regard to types of fat. In women, HR was lowest in the fourth quintile of total fat intake followed by the top quintile; HRs across quintiles were 1.00, 1.03 (0.94–1.11), 1.00 (0.92–1.09), 0.88 (0.81–0.96), and 0.94 (0.86–1.03). Regarding types of fat in women, total mortality was inversely associated with intakes of saturated fatty acids (SFA), monounsaturated fatty acids (MUFA), and polyunsaturated fatty acids (PUFA); the lowest HR was in the top quintile of intake for SFA, MUFA, and PUFA: 0.91 (95% CI, 0.83–1.00), 0.91 (0.83–0.99) and 0.88 (0.80 - 0.97), respectively (trend *P* across quintiles, 0.020, 0.012, and 0.029, respectively). Causes of death other than cancer and cardiovascular disease contributed most to decreases in HRs for total and types of fat. In women, analysis with finer categories revealed that the lowest risk for total mortality appeared at total fat intake of 28% of energy.

**Conclusions:**

Our findings from a large cohort study among populations with relatively low fat intake provide evidence regarding optimal levels of fat intakes.

## Background

Governmental authorities and institutions in developed countries have long recommended reductions in the intake of dietary fat [1-3]. These recommendations are derived mainly from the associations of higher intakes of fat and its components with the risk of cardiovascular disease (CVD), overweight, and cancer, although such associations have not necessarily been supported in subsequent studies [1]. In recent years, greater emphasis has been placed on the reduction of saturated fatty acid (SFA) and *trans*-fatty acid intakes rather than total fat [1,3].

In addition to associations with the risk of specific diseases, relationships to total health may be important in determining optimal lifestyle and medical factors. Total mortality is one of the most concrete indices for total health. Many studies have related total mortality to factors such as alcohol intake [4], sleep [5], and overweight or leanness [6,7], and have proposed acceptable ranges of alcohol consumption, sleep duration, and body mass index (BMI). Examining the associations of fat intake and its components with the risk of total mortality may be a useful basis for formulating dietary recommendations.

Nevertheless, studies relating fat intake in adults to total mortality are scarce. Leosdottir et al. [8] examined the association of intake of total fat and its components with all-cause mortality and reported no clear relationship. Mean intakes of total fat in that study (39.7% and 38.4% of total energy in men and women, respectively), however, were higher compared to the range proposed in dietary guidelines (20-35% of energy) [1-3]. Furthermore, the mean SFA intake (about 16% of energy) exceeded the upper limit recommended in guidelines (7–10% of energy) [1-3].

Nagata et al. [9] recently reported that total fat intake was negatively associated with all-cause mortality in men, but not in women, in a Japanese population with low fat consumption (median, 22.7% and 23.7% of total energy in men and women, respectively). Studies among populations with relatively low fat intake may thus help elucidate optimum intake levels in relation to lowest risk of total mortality. To the best of our knowledge, however, no other study has examined the association between total fat intake and all-cause mortality.

We have been conducting a large cohort study, the Japan Collaborative Cohort (JACC) Study, for the evaluation of cancer risk, sponsored by the Monbu-Kagaku-sho (Ministry of Education, Culture, Sports, Science and Technology) of Japan [10-12] since 1988. This study involves participants throughout Japan, where fat intake is lower compared with Western countries; in 1990, when the baseline survey was undertaken, mean fat intake was 25.3% of total energy [13]. In contrast, total fat intake contributes an average of 33% of energy for men and women in the United States (2007–2010) [14], and 33-39% for men and 33–40% for women in four European countries (2005–2011) [15-18].

We surmised that if the optimum level of fat intake with the lowest risk of mortality exists at a level lower than in Western countries, investigations on the relationship between fat intake and mortality using populations with lower fat intake would prove informative. We therefore examined associations for intakes of total fat and its components with total and major causes of death by analyzing data from the JACC Study.

## Methods

### Study cohort

The baseline survey of the JACC Study was conducted from 1988 through 1990. In that survey, 110,585 male and female inhabitants aged 40 to 79 years completed a questionnaire [10-12]. The JACC Study was a multi-institutional collaborative study in which 24 research institutions and hospitals voluntarily participated. Recruitment of study participants fell to each investigator, who arbitrary defined the area and target population. Participants were enrolled from 45 study areas throughout Japan: 3 towns in Hokkaido district; 5 towns in Tohoku district; 5 towns in Kanto district; 1 city, 3 towns and 2 villages in Chubu district; 8 towns and 2 villages in Kinki district; 1 city and 1 town in Chugoku district; and 4 cities, 9 towns and 1 village in Kyushu district.

In 22 of the 45 study areas, all residents living in a defined target area were regarded as study subjects. In 20 areas, participants in a basic health examination conducted under the Health and Medical Service Law for the Aged were invited to join the study. In 2 areas, study subjects comprised health check-up participants plus volunteers. In 1 area, subjects were recruited from those who undertook the health examination for atomic bomb survivors. The response rate was 83%. Informed consent for participation was obtained individually from each participant, except in a few study areas where consent was obtained at the group level after the aims of the study and the confidentiality of data had been explained to community leaders. The ethics boards of Nagoya University School of Medicine and Hokkaido University Graduate School of Medicine approved the protocol for this investigation, including the procedures used to obtain informed consent.

Potential participants for the present analysis were restricted to 86,401 men and women who lived in 34 study areas where a food frequency questionnaire (FFQ) to estimate food and nutrient intake was included in the baseline questionnaire. Of the 86,401 potential participants, we excluded: 4,678 men and women with a history of cancer, stroke, or myocardial infarction; 22,941 without sufficient responses to the FFQ to estimate nutrient intake (judged by predefined criteria); and 110 with implausibly high or low total energy intakes (<500 or >3,500 kcal/d). As a result, 23,115 men and 35,557 women were eligible for the present analysis.

### Diet and other exposure data

The baseline questionnaire covered lifestyle factors, including dietary habits, smoking and drinking, and physical activity, as well as medical history, education, and height and weight.

The dietary component of the questionnaire included 40 food items [19]. For 33 foods or dishes, we asked about the average intake frequency without specifying portion size. We used the following 5 response choices: almost never; 1–2 times a month; 1–2 times a week; 3–4 times a week; and almost every day. For rice, miso soup (soup of fermented soybean paste with soybean curd, vegetables, and/or seaweed, etc.), and 4 non-alcoholic beverages, we asked about the number of bowls or cups consumed each day. For alcoholic beverages, we inquired about the frequency of consumption and the usual amount on each occasion. Nutrient intakes were computed using the Japanese food composition table [20], assuming standard portion sizes. The supplemented version of the food composition table was employed for n-3 and n-6 polyunsaturated fatty acids (PUFA). Energy-adjusted dietary intakes of nutrients, including total fat, SFA, monounsaturated fatty acids (MUFA), PUFA, and n-3 and n-6 PUFA, and consumption of vegetables and fruit, were calculated by the residual method [21]. For this adjustment for energy, natural logarithms of energy, nutrient, or dietary intakes were used to improve the normality of distributions. Energy intake was not adjusted when computing the PUFA-to-SFA (P/S) ratio.

The FFQ was validated by referring to four 3-day weighted dietary records over a 1-year period as a standard [19]. We reanalyzed data from the validation study to consider skewed distributions of nutrient intakes and within-person variation in intakes [22,23]. De-attenuated correlation coefficients for energy-adjusted intakes between the FFQ and dietary records were 0.51 for total fat, 0.56 for SFA, 0.49 for MUFA, and 0.35 for PUFA. Ratios of median intakes (% of energy [%E]) estimated by the FFQ to those calculated from the dietary records were 0.84, 1.12, 0.80, and 0.68 for total fat, SFA, MUFA, and PUFA, respectively.

### Follow-up

We used population registries from the involved municipalities to determine the vital and residential status of participants. Registration of death is required by the Family Registration Law in Japan and is adhered to nationwide. For logistical reasons, we discontinued the follow-up of subjects who had moved out of their given study area. Causes of death were ascertained by means of a systematic review of death certificates. The underlying cause of death was coded in accordance with the rules of the 10th Revision of the International Statistical Classification of Diseases and Related Health Problems (ICD-10). Cancer was defined using codes C00-C97, CVD using I00-I99, coronary heart disease (CHD) using I20-I25, and stroke using I60-I69.

Follow-up was conducted from the time of the baseline survey through to the end of 2009, except in 6 areas (to the end of 1999, 2003, and 2008 for 3, 1, and 2 areas, respectively). During the study period, 5.8% (*n* = 3,395) of participants in the analytic cohort were lost to follow-up, mostly due to moving away from study areas.

### Statistical analysis

BMI at baseline was calculated from reported height and weight. The linear trend of background characteristics across quintiles of total fat intake was statistically tested using the linear regression model for continuous variables and the Mantel-Haenszel χ^2^ test for categorical variables. In these tests for trend, scores of 0, 1, 2, 3, and 4 were assigned to the successive quintiles.

We counted person-time of follow-up for each participant from the date of filling out the baseline questionnaire to the date of death from any cause, the date of emigration outside the study area, or the end of the follow-up period, whichever came first. Subjects who moved out of their study area were treated as censored cases. Hazard ratios (HRs) with 95% confidence intervals (CIs) for total, cancer, cardiovascular, and other mortality over quintiles of energy-adjusted intakes of total fat, SFA, MUFA, PUFA, and n-3 and n-6 PUFA, and quintiles of P/S ratio (HRs for the second to the highest quintiles versus the lowest) were estimated using proportional hazards modeling [24] by sex. We also computed HRs among male never-drinkers to assess confounding or effect modifications by alcohol consumption, because alcohol accounted for a considerable proportion of energy intake in men. HRs were adjusted for age (as a continuous variable), geographic area (Hokkaido, Tohoku, Kanto, Chubu, Kinki, Chugoku, or Kyushu), educational level (attended school until ≤ 15, 16–18, or ≥ 19 years old), smoking (never-smoker, ex-smoker, or current smoker who smoked < 20, 20–39, or ≥ 40 cigarettes per day), alcohol consumption (never-drinker, ex-drinker, or current drinker who consumed < 1.0, 1.0–1.9, 2.0–2.9, or ≥ 3.0 Japanese standard drinks [23 g of ethanol] per day), BMI (< 20.0, 20.0–24.9, 25.0–29.9, or ≥ 30.0 kg/m^2^), sleep duration (< 6.0, 6.0–6.9, 7.0–7.9, 8.0–8.9, or ≥ 9.0 h per day), daily walking habits (almost none, about 30, 30–60, or ≥ 60 min per day), consumption of vegetables (quintiles 1–5) and fruit (quintiles 1–5), and total energy intake (as a continuous variable). We considered walking time because that was the major physical activity in the study population [25]. Missing values for each covariate were treated as an additional category in the variable and were included in the proportional hazards model. As a basis for the trend tests for HRs over the quintiles, median energy-adjusted fat intake in each quintile was included in the model. We repeated the analyses for total mortality after excluding the first 3 years of follow-up, in which 817 deaths were recorded, from the risk period.

We also attempted further analysis, dividing each of the fourth and top quintiles of total fat intake into lower and upper halves (10% of the cohort) [26] to seek intake levels associated with the lowest risk of total mortality, because a lower HR was found in these two quintiles in women. The reference was set to those with the lowest quintile of intake. In addition, to explicate roles of macronutrients replaced by fat, we added energy-adjusted intake of protein or carbohydrate (as a continuous variable) to the multivariate model for total fat intake. The effect of substituting fat for carbohydrate can be assessed using the multivariate model with energy-adjusted protein intake, and the effect of substituting fat for protein using the model with energy-adjusted intake of carbohydrate [9]. In these models, energy intake is considered to be kept constant because total energy was also incorporated in the model [27]. All *P* values were 2-sided, and all analyses were performed using Statistical Analysis System version 9.1 software (SAS Institute, Cary, NC).

## Results

Median intake of total fat (% of energy) was 10.8–23.3% in men and 13.7–26.8% in women across the quintiles of energy-adjusted intake (Table 1). Intake of protein increased and that of carbohydrate decreased with increasing level of total fat intake. Men and women with higher fat intake tended to consume more vegetables and fruit, to be highly educated, and to have slightly lower BMI, and were less likely to be current smokers. Age correlated positively with fat intake in men, but showed an inverse correlation in women. We found the following associated with increasing fat intake among men: decreasing weight; decreasing trends in proportions of current drinkers and smokers; and increasing trends in the proportions of former drinkers and smokers. Among women, the following were associated with increasing fat intake: decreasing trend in the proportion walking ≥1 h/day; and increasing proportion of current drinkers. Although the trend was significant in women, sleep duration did not materially vary among the fat intake categories.

**Table 1:** Baseline characteristics of subjects by quintile (Q1–Q5) of total fat intake (adjusted for energy intake): 23,115 men and 35,557 women in the JACC Study, 1988-2009

	**Quintiles of total fat intake**					**Trend *P***
	**Q1**	**Q2**	**Q3**	**Q4**	**Q5**	
Men (*n* = 23,115)						
*n*	4,623	4,623	4,623	4,623	4,623	
Age at baseline, y	54.9 ± 9.6	55.0 ± 9.7	55.9 ± 9.8	56.4 ± 10.1	57.7 ± 10.2	<0.001
Total energy intake, kcal/d	1,716 ± 478	1,763 ± 480	1,778 ± 479	1,752 ± 497	1,692 ± 490	0.009
Total fat intake, %E median (IQR)	10.8 (9.4–11.9)	13.9 (13.0–15.0)	16.3 (15.2–17.5)	18.8 (17.6–20.3)	23.3 (21.3–25.8)	< 0.001
SFA	3.0 (2.5–3.5)	4.1 (3.5–4.7)	4.9 (4.3–5.6)	5.8 (5.1–6.6)	7.3 (6.4–8.4)	< 0.001
MUFA	3.3 (2.8–3.7)	4.3 (4.0–4.7)	5.0 (4.7–5.4)	5.9 (5.4–6.4)	7.4 (6.7–8.3)	< 0.001
PUFA	2.6 (2.1–3.1)	3.3 (2.8–3.7)	3.7 (3.3–4.2)	4.2 (3.7–4.6)	5.0 (4.4–5.6)	< 0.001
Protein intake, %E median (IQR)	10.2 (9.3–11.1)	11.9 (11.1–12.7)	13.0 (12.2–13.9)	14.2 (13.2–15.2)	16.0 (14.8–17.5)	< 0.001
Carbohydrate intake, %E median (IQR)	64.2 (56.9–72.4)	61.6 (54.4–68.4)	59.4 (52.8–65.7)	58.0 (51.5–62.9)	53.2 (47.5–58.1)	< 0.001
BMI, kg/m^2^	22.8 ± 3.2	22.7 ± 2.8	22.7 ± 2.7	22.7 ± 3.3	22.6 ± 2.8	< 0.001
Weight, kg	60.8 ± 8.8	60.9 ± 8.9	60.7 ± 8.7	60.5 ± 8.6	60.0 ± 8.7	< 0.001
Sleep duration, h/d	7.4 ± 1.1	7.4 ± 1.0	7.4 ± 1.0	7.4 ± 1.0	7.4 ± 1.1	0.93
College or higher education, %	14.4	16.8	17.5	20.7	23.2	< 0.001
Alcohol consumption						
Current drinkers, %	81.2	79.1	77.4	72.8	64.7	< 0.001
Former drinkers, %	4.1	3.9	5.0	5.9	9.6	< 0.001
Smoking						
Current smokers, %	60.4	55.4	54.0	51.4	49.3	< 0.001
Former smokers, %	22.1	25.4	25.8	25.7	26.0	< 0.001
Vegetable consumption, g/d	80.9 ± 46.6	101.5 ± 50.3	112.0 ± 52.3	123.8 ± 53.5	139.9 ± 57.4	< 0.001
Fruit consumption, g/d	75.0 ± 62.2	94.2 ± 66.0	104.8 ± 68.3	117.0 ± 69.2	128.9 ± 70.8	< 0.001
Walking > = 1 h/d, %	49.4	50.9	50.2	49.5	48.5	0.19
Women (n = 35,557)						
*n*	7,111	7,112	7,111	7,112	7,111	
Age at baseline, y	57.5 ± 10.0	56.4 ± 9.9	56.1 ± 9.9	55.7 ± 9.7	55.6 ± 9.8	< 0.001
Total energy intake, kcal/d	1,429 ± 400	1,447 ± 361	1,451 ± 326	1,463 ± 337	1,401 ± 376	0.002
Total fat intake, %E median (IQR)	13.7 (11.9–15.0)	17.6 (16.8–18.3)	20.1 (19.5–20.7)	22.6 (21.9–23.4)	26.8 (25.3–28.9)	< 0.001
SFA	3.7 (3.1–4.4)	5.2 (4.6–5.9)	6.3 (5.6–6.8)	7.1 (6.5–7.7)	8.6 (7.7–9.7)	< 0.001
MUFA	4.2 (3.6–4.6)	5.4 (5.1–5.7)	6.2 (6.0–6.6)	7.1 (6.8–7.5)	8.6 (8.0–9.3)	< 0.001
PUFA	3.2 (2.6–3.8)	4.0 (3.5–4.5)	4.4 (3.9–4.9)	4.8 (4.3–5.3)	5.5 (4.9–6.2)	< 0.001
Protein intake, %E median (IQR)	12.3 (11.3–13.3)	14.2 (13.4–15.1)	15.3 (14.4–16.2)	16.3 (15.4–17.3)	17.9 (16.7–19.2)	< 0.001
Carbohydrate intake, %E median (IQR)	71.7 (69.7–74.2)	66.6 (65.1–67.9)	63.2 (61.8–64.4)	59.8 (58.3–61.2)	54.3 (51.1–56.6)	< 0.001
BMI, kg/m^2^	23.1 ± 3.3	23.1 ± 4.5	22.9 ± 3.0	22.9 ± 3.0	22.7 ± 3.5	< 0.001
Weight, kg	52.4 ± 8.2	52.7 ± 7.9	52.6 ± 7.7	52.6 ± 7.5	52.4 ± 7.4	0.86
Sleep duration, h/d	7.1 ± 1.1	7.1 ± 1.1	7.1 ± 1.0	7.1 ± 1.0	7.0 ± 1.0	< 0.001
College or higher education, %	7.5	9.1	10.3	11.8	14.8	< 0.001
Alcohol consumption						
Current drinkers, %	21.5	23.2	23.1	24.0	25.1	< 0.001
Former drinkers, %	1.7	1.5	1.4	1.3	1.9	0.73
Smoking						
Current smokers, %	6.5	5.2	4.1	4.1	4.5	< 0.001
Former smokers, %	1.7	1.2	1.3	1.3	1.5	0.39
Vegetable consumption, g/d	101.0 ± 50.7	118.5 ± 51.5	127.7 ± 52.4	137.6 ± 53.1	145.3 ± 54.9	< 0.001
Fruit consumption, g/d	112.6 ± 70.2	133.0 ± 68.9	140.7 ± 67.9	147.2 ± 67.2	149.7 ± 67.4	< 0.001
Walking > = 1 h/d, %	53.3	53.4	51.2	51.2	48.6	< 0.001

Plus-minus values are means ± SD.

During a median follow-up of 19.3 years (interquartile range, 11.4–20.8 years), 11,656 deaths were recorded among 58,672 participants. The most common cause of death was cancer (*n* = 4,241; 36.4%), followed by CVD (*n* = 3,393; 29.1%). Among men (Table 2), we found no clear trend for total mortality across quintiles of fat intakes except for increasing trends in HRs with increasing intakes of PUFA (particularly of n-6 PUFA) and P/S ratio. Excluding the first 3 years of follow-up from the risk period did not essentially alter the findings (data not shown). Significant associations were not detected between fat intakes and total mortality in men in the analysis limited to never-drinkers (*n* = 4,454), although a somewhat reduced risk was seen in the second highest quintile of total fat intake; HRs across the quintiles were 1.00, 1.00 (95% CI, 0.84–1.19), 1.01 (0.84–1.21), 0.92 (0.76–1.11), and 0.98 (0.81–1.18) (trend *P* = 0.58; data not shown for SFA, MUFA, PUFA, n-3 and n-6 PUFA, and P/S ratio).

**Table 2:** Hazard ratios (HRs) for total, cancer, cardiovascular, and other mortality by quintile (Q1–Q5) of energy-adjusted fat intake in men: the JACC Study, 1988-2009 (n = 23,115)

	**No. of deaths**					**HR (95% CI)**^**1**^					**Trend *P***
	**Q1**	**Q2**	**Q3**	**Q4**	**Q5**	**Q1**	**Q2**	**Q3**	**Q4**	**Q5**	
Total fat											
Median of intake, %E						10.8	13.9	16.3	18.8	23.3	
Total mortality	1,178	1,188	1,238	1,236	1,451	1.00	1.03 (0.95–1.12)	1.02 (0.94–1.10)	0.98 (0.90–1.07)	1.07 (0.98–1.17)	0.31
Cancer mortality	454	505	473	491	564	1.00	1.15 (1.01–1.30)	1.03 (0.90–1.17)	1.05 (0.92–1.21)	1.17 (1.02–1.34)	0.12
Cardiovascular mortality	311	302	339	315	398	1.00	0.98 (0.84–1.16)	1.03 (0.88–1.21)	0.91 (0.77–1.08)	1.05 (0.89–1.24)	0.80
Other mortality	413	381	426	430	489	1.00	0.94 (0.81–1.08)	0.99 (0.86–1.14)	0.95 (0.82–1.09)	0.99 (0.85–1.14)	0.87
SFA											
Median of intake, %E						3.0	4.1	4.9	5.8	7.3	
Total mortality	1,323	1,246	1,226	1,230	1,266	1.00	0.96 (0.89–1.04)	0.99 (0.91–1.07)	0.96 (0.88–1.04)	0.98 (0.90–1.06)	0.54
Cancer mortality	527	478	469	494	519	1.00	0.93 (0.82–1.06)	0.95 (0.83–1.08)	0.99 (0.87–1.13)	1.05 (0.92–1.20)	0.45
Cardiovascular mortality	343	336	363	302	321	1.00	0.98 (0.84–1.14)	1.13 (0.97–1.31)	0.89 (0.76–1.05)	0.93 (0.79–1.10)	0.30
Other mortality	453	432	394	434	426	1.00	0.97 (0.85–1.11)	0.93 (0.81–1.07)	0.97 (0.85–1.12)	0.93 (0.80–1.07)	0.32
MUFA											
Median of intake, %E						3.3	4.3	5.0	5.9	7.4	
Total mortality	1,235	1,156	1,226	1,237	1,437	1.00	0.96 (0.88–1.04)	0.98 (0.90–1.07)	0.92 (0.85–1.00)	1.01 (0.93–1.10)	0.99
Cancer mortality	477	469	498	481	562	1.00	1.00 (0.88–1.14)	1.04 (0.91–1.18)	0.96 (0.84–1.10)	1.09 (0.96–1.25)	0.35
Cardiovascular mortality	332	301	315	324	393	1.00	0.92 (0.79–1.08)	0.93 (0.79–1.09)	0.87 (0.74–1.03)	0.99 (0.84–1.17)	0.74
Other mortality	426	386	413	432	482	1.00	0.93 (0.81–1.07)	0.96 (0.83–1.10)	0.92 (0.80–1.06)	0.95 (0.82–1.10)	0.46
PUFA											
Median of intake, %E						2.6	3.3	3.7	4.2	5.0	
Total mortality	1,012	1,078	1,224	1,374	1,603	1.00	1.03 (0.94–1.12)	1.05 (0.96–1.15)	1.08 (0.98–1.18)	1.08 (0.98–1.18)	0.074
Cancer mortality	425	460	491	531	580	1.00	1.04 (0.91–1.19)	1.03 (0.90–1.19)	1.05 (0.91–1.21)	1.03 (0.89–1.19)	0.66
Cardiovascular mortality	246	261	314	370	474	1.00	1.02 (0.85–1.22)	1.06 (0.89–1.26)	1.10 (0.92–1.31)	1.18 (0.99–1.41)	0.044
Other mortality	341	357	419	473	549	1.00	1.02 (0.87–1.18)	1.06 (0.91–1.23)	1.08 (0.92–1.25)	1.05 (0.89–1.23)	0.47
n-3 PUFA											
Median of intake, %E						0.5	0.6	0.8	0.9	1.2	
Total mortality	998	1,091	1,252	1,338	1,612	1.00	0.96 (0.88–1.05)	1.01 (0.93–1.11)	1.01 (0.92–1.10)	1.04 (0.95–1.14)	0.21
Cancer mortality	420	434	491	538	604	1.00	0.93 (0.81–1.06)	0.98 (0.85–1.12)	1.01 (0.88–1.16)	1.02 (0.89–1.18)	0.42
Cardiovascular mortality	254	271	315	378	447	1.00	0.91 (0.77–1.09)	0.95 (0.80–1.13)	1.05 (0.89–1.25)	1.02 (0.86–1.21)	0.36
Other mortality	324	386	446	422	561	1.00	1.05 (0.90–1.22)	1.10 (0.95–1.28)	0.96 (0.82–1.12)	1.08 (0.93–1.26)	0.65
n-6 PUFA											
Median of intake, %E						2.1	2.7	3.2	3.7	4.5	
Total mortality	1,023	1,090	1,236	1,340	1,602	1.00	1.02 (0.94–1.12)	1.08 (0.99–1.18)	1.12 (1.02–1.23)	1.09 (0.99–1.20)	0.022
Cancer mortality	440	472	494	501	580	1.00	1.03 (0.90–1.18)	1.03 (0.90–1.18)	1.02 (0.89–1.18)	1.03 (0.89–1.20)	0.72
Cardiovascular mortality	245	251	326	376	467	1.00	0.96 (0.80–1.15)	1.14 (0.96–1.37)	1.23 (1.03–1.47)	1.18 (0.98–1.42)	0.015
Other mortality	338	367	416	463	555	1.00	1.06 (0.91–1.23)	1.10 (0.94–1.28)	1.15 (0.98–1.35)	1.09 (0.93–1.28)	0.21
PUFA to SFA (P/S) ratio											
Median of P/S ratio						0.49	0.64	0.76	0.91	1.17	
Total mortality	944	1,206	1,243	1,333	1,565	1.00	1.07 (0.98–1.17)	1.03 (0.94–1.12)	1.09 (1.00–1.19)	1.09 (0.99–1.18)	0.083
Cancer mortality	408	488	486	522	583	1.00	1.05 (0.91–1.20)	0.97 (0.84–1.11)	1.01 (0.88–1.15)	0.99 (0.86–1.13)	0.72
Cardiovascular mortality	192	336	333	358	446	1.00	1.38 (1.15–1.66)	1.29 (1.07–1.55)	1.38 (1.15–1.66)	1.39 (1.16–1.66)	0.011
Other mortality	344	382	424	453	536	1.00	0.92 (0.79–1.07)	0.95 (0.82–1.10)	1.03 (0.89–1.19)	1.02 (0.88–1.18)	0.27

^1^Adjusted for age, area, education, smoking, alcohol consumption, BMI, sleep duration, walking, consumption of vegetables and fruit, and total energy intake.

With regard to major causes of death, cardiovascular mortality correlated positively with PUFA (principally n-6 PUFA) intake. This positive association with n-6 PUFA in men was mostly explained by stroke (data not shown). A lower risk of CVD deaths was observed in the lowest quintile of P/S ratio. As for cancer mortality, the HR was significantly elevated in the highest quintile of total fat intake.

**Figure 1:**
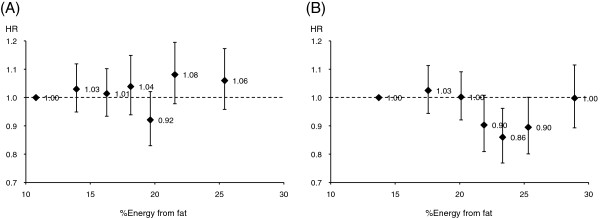
**Hazard ratios (HRs) for total mortality according to total fat intake by sex.** HRs within the first to third quintiles and the lower and upper halves of the fourth and top quintiles of total fat intake (%E) are shown for men (**A**; *n* = 23,115) and women (**B**; *n* = 35,557). The reference group comprised participants with the lowest quintile of fat intake (%E). HRs were adjusted for age, area, education, smoking, alcohol consumption, BMI, sleep duration, walking, consumption of vegetables and fruit, and total energy intake. HRs with 95% confidence intervals (shown as error bars) are plotted against median intake of fat (%E) in each category. The 95% confidence intervals for the second to third quintiles and lower and upper halves of the fourth and top quintiles of intake (%E), from lowest through to highest intake, were 0.95–1.12, 0.93–1.10, 0.94–1.15, 0.83–1.02, 0.98–1.20, and 0.96–1.17 for men, and 0.94–1.11, 0.92–1.09, 0.81–1.01, 0.77–0.96, 0.80-1.00, and 0.89–1.12 for women, respectively.

Among women, total mortality was inversely associated with intakes of total fat, SFA, MUFA, and PUFA (Table 3). The HR was lowest in the second highest quintile of intake for total fat (0.88; 95% CI, 0.81–0.96) followed by the top quintile. This corresponds to a decrease of 131 per 100,000 person-years in the absolute mortality rate in the fourth quintile compared with the lowest quintile. On the other hand, the lowest HR was found in the top quintile of intake for SFA, MUFA, and PUFA. Causes of death other than cancer and CVD contributed most to the decrease in the HR. HRs for total mortality were not substantially changed by excluding the first 3 years of follow-up from the risk period (data not shown).Analysis further dividing the fourth and top quintiles of total fat intake into lower and upper halves revealed a U-shaped association between fat intake and total mortality in women, but not in men (Figure 1). When HRs were plotted against median intake of total fat (%E) in each category, the lowest HR appeared at 23% energy from fat in women.

**Table 3:** Hazard ratios (HRs) for total, cancer, cardiovascular, and other mortality by quintile (Q1–Q5) of energy-adjusted fat intake in women: the JACC Study, 1988-2009 (n = 35,557)

	**No. of deaths**					**HR (95% CI)**^**1**^					**Trend *P***
	**Q1**	**Q2**	**Q3**	**Q4**	**Q5**	**Q1**	**Q2**	**Q3**	**Q4**	**Q5**	
Total fat											
Median of intake, %E						13.7	17.6	20.1	22.6	26.8	
Total mortality	1,284	1,139	1,076	918	948	1.00	1.03 (0.94–1.11)	1.00 (0.92–1.09)	0.88 (0.81–0.96)	0.94 (0.86–1.03)	0.028
Cancer mortality	366	373	364	316	335	1.00	1.07 (0.93–1.25)	1.06 (0.91–1.24)	0.93 (0.79–1.09)	1.01 (0.87–1.19)	0.68
Cardiovascular mortality	430	371	356	278	293	1.00	1.05 (0.91–1.21)	1.07 (0.92–1.24)	0.87 (0.75–1.03)	0.97 (0.83–1.14)	0.34
Other mortality	488	395	356	324	320	1.00	0.97 (0.85–1.11)	0.90 (0.78–1.04)	0.85 (0.73–0.98)	0.87 (0.75–1.01)	0.018
SFA											
Median of intake, %E						3.7	5.2	6.3	7.1	8.6	
Total mortality	1,426	1,144	1,061	936	798	1.00	0.97 (0.90–1.05)	0.97 (0.90–1.06)	0.91 (0.84–1.00)	0.91 (0.83–1.00)	0.020
Cancer mortality	397	381	336	360	280	1.00	1.06 (0.92–1.22)	0.96 (0.83–1.11)	1.06 (0.92–1.24)	0.92 (0.79–1.09)	0.54
Cardiovascular mortality	484	363	363	264	254	1.00	0.97 (0.84–1.11)	1.07 (0.92–1.23)	0.85 (0.72–0.99)	0.99 (0.84–1.16)	0.46
Other mortality	545	400	362	312	264	1.00	0.91 (0.80–1.04)	0.91 (0.79–1.05)	0.84 (0.73–0.97)	0.84 (0.72–0.98)	0.009
MUFA											
Median of intake, %E						4.2	5.4	6.2	7.1	8.6	
Total mortality	1,337	1,118	1,038	948	924	1.00	0.98 (0.90–1.06)	0.95 (0.88–1.04)	0.91 (0.83–0.99)	0.91 (0.83–0.99)	0.012
Cancer mortality	374	371	363	320	326	1.00	1.05 (0.91–1.22)	1.06 (0.91–1.23)	0.94 (0.81–1.10)	0.99 (0.84–1.16)	0.56
Cardiovascular mortality	453	359	334	293	289	1.00	0.98 (0.85–1.13)	0.98 (0.84–1.13)	0.92 (0.79–1.08)	0.94 (0.80–1.10)	0.32
Other mortality	510	388	341	335	309	1.00	0.91 (0.80–1.04)	0.84 (0.73–0.97)	0.88 (0.76–1.02)	0.82 (0.70–0.95)	0.006
PUFA											
Median of intake, %E						3.2	4.0	4.4	4.8	5.5	
Total mortality	1,038	923	1,039	1,144	1,221	1.00	0.91 (0.83–1.00)	0.96 (0.88–1.06)	0.95 (0.87–1.04)	0.88 (0.80–0.97)	0.029
Cancer mortality	343	306	352	380	373	1.00	0.88 (0.75–1.03)	0.95 (0.81–1.11)	0.95 (0.81–1.12)	0.86 (0.72–1.01)	0.17
Cardiovascular mortality	329	283	344	363	409	1.00	0.90 (0.77–1.06)	1.03 (0.87–1.21)	0.96 (0.81–1.13)	0.93 (0.79–1.10)	0.59
Other mortality	366	334	343	401	439	1.00	0.95 (0.82–1.11)	0.91 (0.78–1.07)	0.93 (0.80–1.09)	0.85 (0.73–1.00)	0.061
n-3 PUFA											
Median of intake, %E						0.6	0.8	0.9	1.1	1.3	
Total mortality	943	973	1,088	1,075	1,276	1.00	0.95 (0.87–1.04)	0.95 (0.87–1.05)	0.91 (0.83–1.00)	0.92 (0.84–1.01)	0.053
Cancer mortality	317	333	351	363	388	1.00	0.97 (0.83–1.13)	0.93 (0.79–1.09)	0.91 (0.77–1.07)	0.88 (0.74–1.04)	0.092
Cardiovascular mortality	298	296	388	337	408	1.00	0.90 (0.77–1.07)	1.06 (0.91–1.24)	0.91 (0.77–1.07)	0.92 (0.78–1.09)	0.36
Other mortality	328	344	349	375	480	1.00	0.96 (0.82–1.12)	0.87 (0.74–1.01)	0.89 (0.76–1.05)	0.95 (0.81–1.11)	0.42
n-6 PUFA											
Median of intake, %E						2.6	3.3	3.8	4.3	5.0	
Total mortality	1,003	922	1,000	1,181	1,249	1.00	0.94 (0.86–1.03)	1.00 (0.91–1.10)	1.00 (0.91–1.10)	0.89 (0.81–0.98)	0.065
Cancer mortality	328	324	342	390	368	1.00	0.98 (0.84–1.15)	1.01 (0.86–1.19)	1.05 (0.89–1.23)	0.89 (0.75–1.05)	0.33
Cardiovascular mortality	312	281	314	391	429	1.00	0.93 (0.79–1.10)	1.02 (0.86–1.21)	1.04 (0.88–1.23)	0.95 (0.81–1.13)	0.92
Other mortality	363	317	344	400	452	1.00	0.89 (0.76–1.04)	0.95 (0.81–1.11)	0.91 (0.78–1.06)	0.83 (0.71–0.97)	0.032
PUFA to SFA (P/S) ratio											
Median of P/S ratio						0.48	0.61	0.71	0.85	1.12	
Total mortality	736	860	1,070	1,170	1,529	1.00	0.97 (0.88–1.08)	1.01 (0.92–1.12)	1.03 (0.93–1.13)	1.02 (0.93–1.12)	0.40
Cancer mortality	286	303	340	421	404	1.00	0.90 (0.77–1.07)	0.90 (0.76–1.06)	1.08 (0.92–1.26)	0.88 (0.75–1.04)	0.49
Cardiovascular mortality	207	270	367	342	542	1.00	1.07 (0.89–1.28)	1.17 (0.98–1.40)	0.99 (0.83–1.19)	1.12 (0.94–1.32)	0.48
Other mortality	243	287	363	407	583	1.00	0.98 (0.82–1.16)	1.01 (0.85–1.20)	1.03 (0.87–1.21)	1.08 (0.92–1.26)	0.19

^1^Adjusted for age, area, education, smoking, alcohol consumption, BMI, sleep duration, walking, consumption of vegetables and fruit, and total energy intake.

In analyses with further adjustment for energy-adjusted intake of protein or carbohydrate, a slightly lower HR for total mortality tended to be seen in the fourth quintile of total fat intake among men, but did not reach the level of statistical significance (*P* > 0.10). HRs across quintiles were 1.00, 1.01 (95% CI, 0.92–1.11), 0.99 (0.89–1.09), 0.94 (0.84–1.06), and 1.02 (0.89–1.17) in the protein-adjusted model (trend *P* = 0.88), and 1.00, 1.02 (95% CI, 0.93–1.11), 0.99 (0.91–1.09), 0.95 (0.86–1.05), and 1.02 (0.90–1.16) in the carbohydrate-adjusted model (trend *P* = 0.76). Among women, the association between total fat intake and all-cause mortality remained similar even after such adjustment, but was somewhat attenuated after adjustment for protein. The linear trend was not significant in either the protein- or carbohydrate-adjusted model. Respective HRs were 1.00, 1.04 (95% CI, 0.95–1.14), 1.03 (0.92–1.14), 0.91 (0.81–1.02), and 0.98 (0.86–1.12) in the protein-adjusted model (trend *P* = 0.32), and 1.00, 1.02 (95% CI, 0.93–1.13), 1.00 (0.89–1.13), 0.88 (0.76–1.02), and 0.94 (0.78–1.14) in the carbohydrate-adjusted model (trend *P* = 0.29).

## Discussion

In this large, community-based, prospective cohort study, we found overall inverse associations of total mortality with intakes of total fat, SFA, MUFA, and PUFA in women, particularly among those aged ≥ 60 years, but not in men. In women, detailed analysis revealed a U-shaped association between total fat intake and total mortality; the lowest risk appeared within the second highest quintile of energy-adjusted fat intake. Regarding major causes of death in men, an upward trend in the HR for CVD mortality was detected with increasing PUFA intake and the HR for cancer mortality was elevated in the highest quintile of total fat intake. Among women, causes of death other than cancer and CVD contributed most to the decreases in HR associated with increasing fat intake.

In women, the lowest HR for total mortality appeared at the upper half of the fourth quintile of total fat intake, at about 23%E. Given the underestimation of the intake (%E) with the FFQ for the JACC Study (~16%), the lowest risk might be associated with a higher intake of about 28%E. This falls within the acceptable range recommended by the Food and Agriculture Organization of the United Nations (FAO) consultation [1] and the United States Department of Agriculture (USDA) guideline (20–35%E) [3], but may exceed the upper limit (25%E) proposed in dietary reference intakes for Japanese individuals of 30 years or older [2]. If the lowest risk for female total mortality really is near 28%E of total fat intake, however, our findings may not be in line with those of Leosdottir *et al.*[8]. They did not find any increase in risk among women who consumed total fat ≥ 35%E compared with those in the reference group with a mean of 30.8%E.

With regard to cancer, the World Cancer Research Fund (WCRF) and the American Institute for Cancer Research (AICR) extensively reviewed the literature and concluded that there is no convincing or probable evidence for significant effects of dietary fats on any type of cancer [28]. In the present study, however, the HR for male cancer mortality was significantly increased by 17% in the top quintile of total fat intake (Table 2), although no linear trend was apparent. The elevated risk was predominantly observed for colorectal cancer; we did not present this finding in the Results section because detailed analyses by cancer site were outside of the scope of this study, and the relevant data from the JACC Study have yet to be published. Nevertheless, the systematic review by the WCRF and AICR [28] mentioned that there is a limited amount of fairly consistent evidence suggesting that consumption of foods containing animal fats is a contributing causal factor for colorectal cancer. The role of dietary fat in the development of colorectal cancer may thus help to explain the higher HR for total cancer mortality associated with higher intake of total fat.

For SFA, the lowest HR in women was found in the highest quintile of intake, which would correspond to 7.7%E considering the overestimation with the FFQ. Although whether the risk of total mortality rises with further increase in SFA intake was not clear, this percentage is consistent with saturated fat intake recommendations by the FAO consultation (<10%E) and USDA (<10 or 7%E). The dietary reference intake for Japanese (4.5–7%E) is lower. Both the FAO consultation and USDA guideline recommend replacement of excessive SFA with PUFA [1,3]. This recommendation is mainly based on evidence that replacing dietary SFA with PUFA is associated with a decreased risk of CHD in both randomized controlled trials and cohort studies [29]. In contrast, replacing SFA with carbohydrate or MUFA has been correlated with an increased risk of CHD in observational cohorts [29]. Nevertheless, our findings regarding P/S ratio did not support the recommendation; in the present study, the risk for cardiovascular death increased at a higher P/S ratio in men.

In a detailed analysis specific to CVD from the JACC Study [30], SFA intake was inversely associated with mortality from stroke, but was not correlated with mortality from CHD, again consistent with substitution models replacing SFA with carbohydrate [30]. The risk for overall CVD death was decreased with increasing intake of SFA in that study. The inverse correlation of SFA intake with CVD risk was much weaker in the current study compared with the previous analysis [30]. However, this may be explicable principally by differences between the previous and present analyses in terms of the statistical analyses, including the covariates incorporated in the models. In particular, we did not include other fatty acids in the model, while the previous study included both n-3 and n-6 PUFA [30]. This is because we did not intend to adjust for the effects of other fatty acid intakes correlated with SFA intake in the daily diet. When we applied a simple age- and sex-adjusted model as in the similar analysis in the previous study [30], an inverse association was clearly apparent; HRs for CVD across quintiles of SFA intake were 1.00, 0.89 (95% CI, 0.81–0.99), 0.91 (0.82–1.00), 0.77 (0.69–0.85), and 0.74 (0.67–0.83) (trend *P* < 0.001). This indicates that the SFA-CVD association was essentially comparable between the present and previous analyses. SFA intake tended to be associated, although not significantly, with a decreased risk of stroke in a meta-analysis of cohort studies [31]. Regarding the role of SFA in relation to all-cause mortality when used to replace macronutrients, Nagata et al. showed an association between high SFA intake and higher total mortality among women in a model substituting SFA for carbohydrates [9].

In women, the inverse association between SFA intake and mortality was most evident for those causes of death other than cancer and CVD. Those causes mainly comprised injury (ICD-10: S00-T98) and pneumonia (ICD-10: J12-J18), accounting for 19.4% and 15.9% of all other causes of female deaths (*n* = 1,883), respectively. Low serum cholesterol levels have been associated with external-cause mortality such as suicide and accidents in prospective studies [32-34]. Such results may support the current findings, because the increased SFA intake raises serum cholesterol levels [29] and therefore might decrease the risk of external-cause mortality. In Japan, suicide and accidents accounted for almost all external causes of death (injury deaths), at 53.3% and 41.7%, respectively, in 1999, as the midpoint of the follow-up period for the present study [35]. In addition to external causes of death, community-acquired pneumonia has been associated with low serum cholesterol in high-risk populations [36]. Fat insufficiency may disturb the absorption of fat-soluble vitamins including vitamins A and E [1-3], which are required to maintain resistance to infectious diseases [37].

Only a few studies have directly correlated MUFA intake with all-cause mortality. In the Italian Longitudinal Study on Aging, higher MUFA intake was associated with decreased total mortality (HR for one standard deviation (SD) increment, 0.81; 95% CI, 0.66–0.99) [38], which may corroborate the inverse association among women in the current study. Alternatively, the Mediterranean diet has often been associated with decreased risk of all-cause death, cancer, and CVD [39]. This may support our finding because the Mediterranean diet is rich in MUFA. However, other dietary factors in the Mediterranean diet, such as vegetables and fruit, have to be considered [39]. Since dietary MUFA intake was very strongly correlated with SFA intake, examining the effect of dietary MUFA independently of SFA using multivariate models was difficult; the correlation coefficient for energy-adjusted intake was 0.86 in men and 0.85 in women in the analytic cohort.

PUFA intake correlated negatively with total mortality in women, which appeared to be due to a decreased risk of cancer death associated with a higher intake of n-3 PUFA and a decreased risk of death from other causes associated with a higher intake of n-6 PUFA. Marine n-3 PUFA was associated with a lower risk of breast cancer in a meta-analysis of cohort studies [40]. Although no protective effect of n-3 fats against cancer was shown in a systematic review [41], typical fish intake is much higher in Asian populations, including Japanese, than in Western populations [40]. One possibility, therefore, is that preventive effects of n-3 PUFA are suggested in studies from Japan. One study related linoleic acid intake to a lower risk of community-acquired pneumonia [42], which may partly explain the association between decreased risk of death from other causes and higher intake of n-6 PUFA.

We did not expect the elevated risk of male cardiovascular mortality associated with higher intake of n-6 PUFA, as several studies have shown inverse associations between n-6 PUFA intake and CVD [43]. This finding could represent a chance phenomenon, since it was not observed in women. The role of dietary n-6 PUFA in the development of CVD, however, remains controversial [44,45], and this study may provide additional data for discussion. Of note, the increased risk of male CVD death was principally due to stroke instead of CHD. Further investigations may therefore need to focus on stroke.

The JACC Study previously reported an inverse association between n-3 PUFA intake and CVD mortality [46] that was not clear in the current analysis. As mentioned in the discussion on SFA and CVD, this apparent inconsistency may be attributable to differences between the previous and current analyses in terms of the covariates included in multivariate models, especially dietary intakes of SFA and n-6 PUFA. When age- and sex-adjusted HRs were estimated as in the preceding study [46], we found an inverse (though not strong) association similar to that in the previous study; HRs for CVD across quintiles of n-3 PUFA intake were 1.00, 0.89 (95% CI, 0.80–1.00), 0.97 (0.87–1.09), 0.98 (0.88–1.09), and 0.89 (0.80–1.00) (trend *P* = 0.21). Regarding investigations on fat and cancer in the JACC Study, the higher serum level of n-3 PUFA was associated with decreased risk of male colorectal cancer [47] and higher dietary intakes of fish fat and long-chain n-3 fatty acids were correlated with a reduced risk of female breast cancer [48]. Those studies, however, cannot be directly compared with our own because of the large difference in exposure assessment; they used serum fatty acids or specific fat/fatty acids (e.g., fish fat, long-chain n-3 fatty acids) instead of dietary total n-3 PUFA. Furthermore, those studies defined incidence of cancer as endpoints, while we adopted cancer deaths.

In Europe and the United States, high fat intake is associated with unhealthy living and unfavorable social class, represented as low intakes of fruit, vegetables and fiber, and increased smoking and alcohol intake on a population level [49]. Conversely, a higher fat intake was associated with higher education, less smoking and male alcohol consumption, and higher consumption of fruit and vegetables in the current study (Table 1). One study based on nationwide surveys in Japan reported an increasing intake of fat (%E) with increasing household expenditure in men and women [50]. Household expenditure also correlated positively with vitamin C and fiber intakes in that study. This suggests that individuals in a higher socioeconomic status in Japan consume not only more foods considered good for health (e.g., vegetables and fruit), but also more fat-rich foods, as seen in our study. Similar correlations between nutrient intakes and income or educational level have also been observed in Chinese women [51].

We therefore considered background characteristics and lifestyle factors including consumption of vegetables and fruit in participants as much as possible from the available data in our statistical analyses. Residual confounding, however, might have affected the results, and the differences in findings regarding fat and mortality among various populations may be partly attributable to differences in characteristics related to fat intake among populations.

No associations between fat intake and total mortality were apparent in men. This is inconsistent with findings from the Takayama Study among Japanese, which showed an inverse correlation with risk of all-cause mortality for total fat only in men [9]. For comparison, we tried to further adjust for protein intake as performed in that study [9], but no significant correlation appeared in men.

The difference in alcohol consumption between sexes may be relevant at least in our cohort, and possibly among Japanese in general. Alcohol consumption contributes to energy intake and therefore decreases the percent of energy derived from fat, even if fat intake remains the same. Percentages of non-alcohol energy were not applied in the present study because dietary guidelines do not explicitly exclude alcohol energy from the denominator [1-3]. In the current cohort, women consumed only 1.7 g (12 kcal) per day of alcohol on average, whereas men drank 25.4 g (180 kcal) per day. We did not identify significant trends between fat intake and total mortality in men, even in the analysis limited to never-drinkers. Sex differences in our population are thus not well explained and should be examined in further investigations.

A tendency toward a somewhat reduced risk, however, was observed in the second highest quintile of total fat intake (HR, 0.92) among male never-drinkers, although this was not significant, partly due to the rather small sample size (*n* = 4,454). Larger studies of non-drinkers may also be warranted to evaluate effect modification or possible residual confounding by alcohol intake.

Similar sex differences were also found for SFA, MUFA, and PUFA in the present study; risk of total mortality was decreased with increasing intakes of SFA, MUFA and PUFA in women, whereas the risk was somewhat elevated with increasing PUFA intake in men. These findings, however, are not in line with preceding studies. Leosdottir et al. [8] reported a positive correlation between MUFA intake and total mortality, particularly in men, while Nagata et al. [9] found an inverse correlation of PUFA and a positive correlation of SFA with all-cause mortality in men and women, respectively. Further investigations are required before any conclusions can be drawn regarding differences in the effects of fatty-acid groups by sex.

The strengths of the current study include the prospective design, large sample size, long follow-up, and multivariate adjustments for potential confounding factors. The design enabled us to define death as an endpoint, and more than 11,000 events made detailed analysis possible. Moreover, the relatively lower level of fat intake among samples provided a unique opportunity to examine associations of fat intake with total mortality.

Some methodological limitations to our study warrant discussion. First, we used a simple FFQ with only 40 food items. Total energy intake may thus have been underestimated because of the foods not covered by the FFQ. However, the FFQ was validated by referring to dietary records, and the over- or underestimation of intakes (%E) of total fat and its components were adjusted using information from the validation study. We could not estimate the intake of *trans*-fatty acids with this FFQ. Although the mean intake in Japan [52,53] is reported to be lower than the proposed upper limit (1% of total energy) [1], some Japanese, particularly women, might have a higher intake of *trans*-fatty acids [53]. Second, we excluded substantial numbers of participants (*n* = 22,941) because of insufficient dietary information. The omitted participants were older and more likely to be men compared with those included in the analysis (mean age ± SD, 60.9 ± 9.8 vs. 56.1 ± 9.9 years; men, 45.7% vs. 39.4%). Other background characteristics, however, were quite similar between the 2 groups of participants, suggesting that these exclusions did not lead to any major selection bias. Third, fat intakes were assessed only once, at baseline. Fourth, the self-reporting of body weight and energy/nutrient intake might have led to bias. Underreporting of energy intake has been reported as more pronounced among women, overweight subjects and those with higher fat intake in European populations [54]. In Asian populations, two studies reported that overweight or obese men and women (BMI ≥25 kg/m^2^) tended to underestimate their weight and/or BMI [55,56]. Finally, because the present study was conducted among Japanese and the health effects of dietary fat may vary among populations with different genetic backgrounds [57], extrapolation to other populations must be made with caution.

## Conclusions

A U-shaped association was found between total fat intake and all-cause mortality in women. The risk for female mortality decreased with increasing intakes of SFA, MUFA, and PUFA through the top quintile of intakes. Our findings from a large cohort study among populations with relatively low fat intake will provide evidence regarding optimal levels of fat intake.

## Competing interests

The authors declare that they have no competing interests.

## Authors’ contribution

KW, MN, CD, HI, and AT designed the study. CD and HI developed methods to estimate dietary intakes of nutrients. HI and AT coordinated the research. KW analyzed the data and drafted the manuscript. All authors read and approved the final manuscript.
